# Bounds on the Average Sensitivity of Nested Canalizing Functions

**DOI:** 10.1371/journal.pone.0064371

**Published:** 2013-05-31

**Authors:** Johannes Georg Klotz, Reinhard Heckel, Steffen Schober

**Affiliations:** 1 Institute of Communications Engineering, Ulm University, Ulm, Germany; 2 Department of Information Technology and Electrical Engineering, ETH Zurich, Zurich, Switzerland; UMIT, Austria

## Abstract

Nested canalizing Boolean functions (NCF) play an important role in biologically motivated regulatory networks and in signal processing, in particular describing stack filters. It has been conjectured that NCFs have a stabilizing effect on the network dynamics. It is well known that the average sensitivity plays a central role for the stability of (random) Boolean networks. Here we provide a tight upper bound on the average sensitivity of NCFs as a function of the number of relevant input variables. As conjectured in literature this bound is smaller than 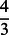. This shows that a large number of functions appearing in biological networks belong to a class that has low average sensitivity, which is even close to a tight lower bound.

## Introduction

Boolean networks play an important role in modeling and understanding signal transduction and regulatory networks. Boolean networks have been widely studied under different point of views, e.g. [1]–[3]. One line of research focuses on the dynamical stability of randomly created networks. For example, random Boolean networks tend to be unstable, if the functions are chosen from the set of all Boolean functions with average number of variables (average *in-degree*) larger than two [4]. This can be attributed to the fact that the expected *average sensitivity* of random Boolean functions with an in-degree 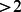 is larger than one. The expected average sensitivity is an appropriate measure for the stability of random Boolean networks [5], [6].

If only functions from certain classes are chosen, stable behavior can be achieved for higher in-degrees. For instance, canalizing and nested canalizing functions, introduced in [7], [8], have been conjectured [9] to have a stabilizing effect on network dynamics. In [10] it has been shown that Boolean networks can be stable, even if the average in-degree is high. Interestingly, studies of regulatory network models in Biology have shown that a large number of their functions are canalizing [11]–[16]. Canalizing functions are also important for the construction of stack filters used in signal processing [17].

A Boolean function 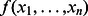 is *canalizing* in variable 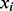, if 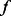 is constant as long as 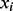 is set to its *canalizing* value. Nested canalizing functions are canalizing functions, whose restriction to the non-canalizing value is again a canalizing function and so on (a precise definition is given later). In this paper we analyze nested canalizing functions (NCFs), in particular their average sensitivities. The notion of sensitivity was first introduced by Cook et al. [18]. It was applied later to Boolean functions [19] and can be viewed as a measure of the impact of a permutation of the input variables on the output of the function. The average sensitivity was investigated in [20] in the context of monotone Boolean functions. An upper bound for locally monotone functions was presented in [15]. Here we give a tight upper bound on the average sensitivity of NCFs. Our result shows that the average sensitivity of NCFs is always smaller than 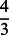 as conjectured in [21]. We further provide a recursive expression of the average sensitivity and the zero Fourier coefficient of a NCF. Finally we discuss and compare our new bounds to bounds in literature.

Our main tool is the Fourier analysis [22], [23] of Boolean functions, which is introduced in Section *Notation, Basic Definitions and Fourier Analysis of Boolean Functions*, where we also address further concepts needed. In Section *Nested Canalizing Functions* spectral properties of canalizing and NCFs are broached. Additionally we discuss functions, in which all variables are most dominant, as they turn out to minimize the average sensitivity. In Section *Average Sensitivity* new bounds on the average sensitivity are presented based on a recursive expression of the average sensitivity of NCFs. We conclude with a discussion of the results and some final remarks.

## Methods

### Notation, Basic Definitions and Fourier Analysis of Boolean Functions

A Boolean function (BF) 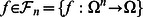 with 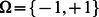 maps n-ary input tuples to a binary output. Note that we choose the 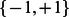-representation of the Boolean states instead of the 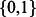-representation, since it will turn out to be advantageous as it simplifies our calculations in the Fourier domain. However, our results apply for all binary alphabets 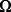.

In general not all input variables have an impact on the output, i.e., are relevant.

**Definition 1** [*21*] *A variable* 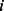 *is relevant to a BF* 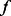, if there exists an 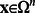 such that
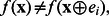

*where* 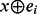 *is the vector obtained from* 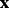 by flipping its 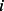-th entry.

*Further we define* 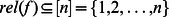 *as the set containing all relevant variables of* 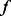.

#### Fourier analysis of boolean functions

In this section we recall basic concepts of Fourier analysis of BFs and some results from [15] concerning restrictions of BFs. Let 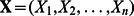 be a random variable uniformly distributed on 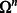, i.e.,
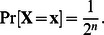



For 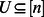 we define the basis functions 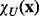 by
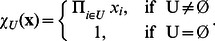
(1)


Note that for 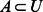 and 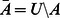.
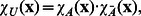
which follows directly from the definition of 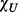 (Eq. (1)).

Any BF 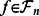 can be represented by its Fourier-expansion [22], [23], i.e.,
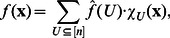
(2)where 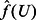 are the Fourier coefficients, given by



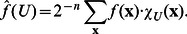
(3)
**Example 1** *Table 1* *and* *Table 2* *contain the truth-table representation and the polynomial representation, i.e., Eq. (2), of AND, OR, and XOR.*

**Table 1:** Truth-table representation.

*x_1_*	*x*_2_	AND	OR	XOR
+1	+1	+1	+1	+1
+1	–1	+1	–1	–1
–1	+1	+1	–1	–1
–1	–1	–1	–1	+1

**Table 2:** Polynomial representation (Eq. (2) ).

AND	OR	XOR
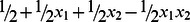	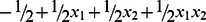	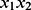

**Remark 1** *The polynomial representation in the previous example is different to the one used in* [*21*] *, where the variables 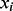 are defined over GF(2), i.e. 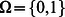, where addition (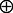) and multiplication are defined modulo 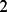. In this case, the AND function becomes 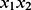, the OR function is given by 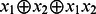 and XOR by 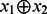.*

#### Restrictions of boolean functions

We call a function 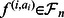 a restriction of 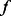, if it is obtained by setting the 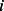-th input variable of 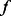 to some constant 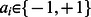. Every BF can be decomposed in two unique restricted functions for each relevant variable, as stated by the following proposition:

**Proposition 1** *For any* 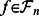 *and each* 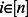 *there exist unique functions* 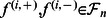 *, with* 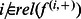 *and* 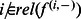 *, such that.*

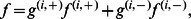

*where the functions* 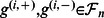 *are given by*



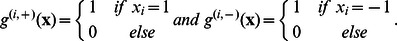



Next we characterize the Fourier coefficients of 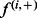 and 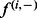.

**Proposition 2** [*15*] *Let* 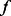 *be a BF in* 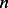 variables. The Fourier coefficients of 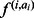 are given by
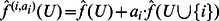

*where* 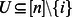.

The reverse relation, i.e., the composition of a BF by two restricted functions, is described in terms of Fourier coefficients by the following proposition:

**Proposition 3** [*15*] *The Fourier coefficients of a *BF 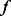 *with uniform distributed input variables can be composed in terms of the Fourier coefficients of its two restricted functions* 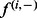 *and* 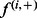 *according to*

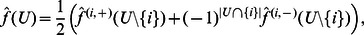

*or*



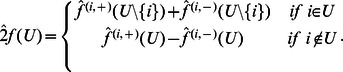



An immediate corollary of Proposition 3 shows that the zero coefficient of a function only depends on the zero coefficients of the restricted functions:

**Corollary 1** *The zero Fourier coefficient of any Boolean function* 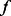 *can be written as:*

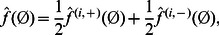
(4)
*where* 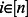 *is the index of some variable.*

If we restrict a function to more than one variable, namely to a set of variables 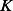, we denote the restricted function with 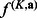, where 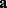 is a vector containing the values to which the function is restricted. The Fourier coefficients of 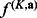 are given by the following proposition:

**Proposition 4** [*15*] *Let* 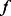 *be a Boolean function and* 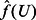 *its Fourier coefficients. Furthermore, let* 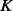 *be a set containing the indices* 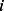 *of the input variables* 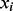 *, which are fixed to certain values* 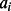. *The Fourier coefficients of the restricted function* 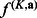 *are then given as:*

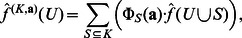

*where* 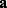 *is the vector with entries* 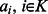.

### Nested Canalizing Functions

In order to define NCFs we first need the following definition:

**Definition 2** *A BF* 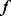 *is called* 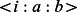 *canalizing, if there exists a canalizing variable* 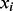 *and a constant* 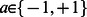 *, such that.*

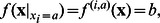

*for all* 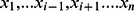 *, where* 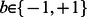 *is a constant.*

Hence, 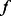 is canalizing in variable 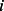, if the decomposition according to Proposition 1 results in either 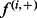 or 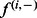 being a constant function.

As shown in [15] the Fourier coefficients of a canalizing function satisfy
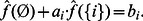
(5)


A NCF can be described recursively as a canalizing function, whose restriction is again a NCF or more formally:

**Definition 3** *For* 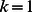 *and* 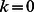 *any BF with* 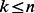 *relevant variables is a NCF. For* 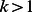 *a BF is a NCF, if there exists at least one variable* 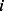 *and constants* 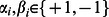 *, such that* 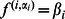 *and* 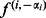 *is a NCF with* 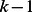 *relevant variables.*

Let 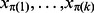 be the variable order for which a NCFs fulfills the properties from Definition 3, then we call, following [21], such a function 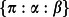 nested canalizing.

As shown in [15] 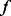 is 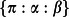 nested canalizing, if for all 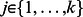.
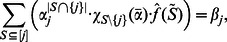
where 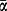 is a vector containing all negated 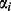, i.e. 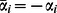 and 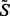 is a set, which is retrieved by applying the permutation 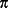 to the elements of 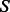.

In order to illustrate the spectral properties of a NCF, consider the following example:

**Example 2** *Let* 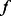 *be a* 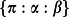 *NCF with* 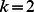 *relevant variables and* 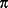 *such that* 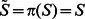 *, then*

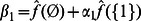


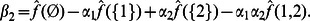



#### Properties of nested canalizing functions

In this section we state some properties of NCF. First we address most dominant variables, which are defined as follows:

**Definition 4** *[21, Def 4.5] Variable* 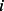 *is called a most dominant variable of* 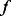 *, if there exists a permutation* 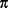 *, such that* 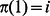 *, for which* 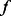 *is* 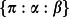 *nested canalizing.*

The set of most dominant variables has an impact on a number of Fourier coefficients, which is summarized in the following proposition.

**Proposition 5** *Let* 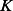 *be the set of most dominant variables of a* 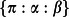 *NCF* 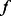 *. Then the absolute values of the corresponding Fourier coefficients are all equal, i.e.,* 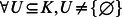.
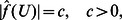

*or, more general,*

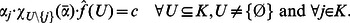
(6)Furthermore,



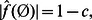

*and*



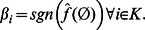



*Proof.* The proof for the zero and first order coefficients, i.e., 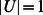 and 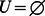, follows directly from Eq. (5). We can hence use Eq. (6) as an induction hypothesis for coefficients with order smaller than 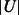. We show next that as a result Eq. (6) is also valid for coefficients with order 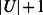.

Using
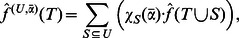
and that 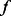 is canalizing in any variable 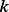, it follows that every restriction of 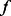 must also be canalizing in variable 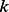, i.e., 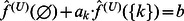, we get:



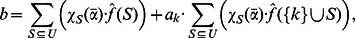


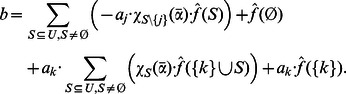



Using Eq. (5) and the induction hypothesis, we get
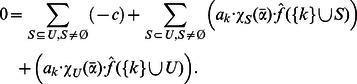



We again use that Eq. (6) holds for all 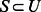, i.e. 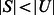 and, hence:
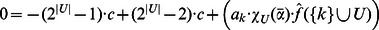


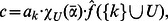
which concludes the proof.

For the special case, in which all variables are most dominant, we derive the following corollaries:

**Corollary 2** *Let* 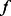 *be a* 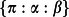 *NCF with* 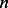 *variables of which* 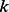 *are relevant. All relevant variables are most canalizing, if the Fourier coefficients satisfy*

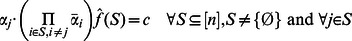
(7)
*with*



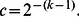
(8)
*Proof.* Eq. (7) follow, directly from Proposition 5, while Eq. (8) follows from Parseval's theorem.

Corollary 2 can easily be rewritten as:

**Corollary 3** *Let* 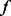 *be a* 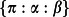 *NCF with* 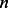 *variables of which* 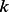 *are relevant. All variables are most canalizing, if the absolute values of the Fourier coefficients fulfill the following conditions,*

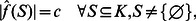
(9)

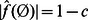

*with*



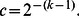
(10)
**Corollary 4** *Let* 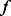 *be a* 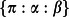 *NCF with* 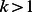 *relevant input variables. All variables are most canalizing and* 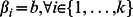 *. All such NCFs are completely described by* 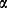 *and* 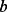 *and hence there are* 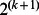 *such functions.*

*Proof.*. The statement follows directly from the previous corollary.

Interestingly, we can describe the zero coefficients for NCFs in a recursive manner:

**Corollary 5** *The zero coefficient of a* 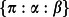 *NFC* 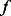 *can be recursively written as:*

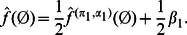



*Proof.* Follows directly from Corollary 1.

Further, the zero coefficient is upper bounded as shown by following proposition:

**Proposition 6** *The absolute value of the zero coefficient of a NCF* 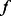 *with* 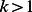 *relevant input variables can be bounded as:*

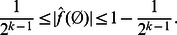



*Proof.* First, we prove the right hand side: Using the triangle inequality we get from Corollary 5:
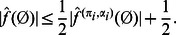



Obviously the zero coefficient of a function with only one relevant variable 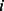 is zero. The proposition now follows by induction. The left hand side can be easily shown using the inverse triangle inequality and induction.

As seen in Corollary 3, a NCF, whose variables are most dominant, fulfills the upper bound in Proposition 6 with equality. The following proposition follows directly from Corollary 5:

**Proposition 7** *The absolute value of the zero coefficient of a NCF* 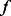 *with* 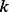 *relevant variables and alternating* 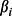 *, i.e., with* 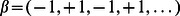 *or* 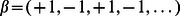 *is given as:*

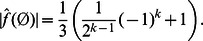



### Average Sensitivity

Before addressing the average sensitivity we first need to define the influence of a variable, which is a measure of the impact of a perturbation of this variable's value.

**Definition 5** *(* *[24]*, *[25]* *) The influence of variable* 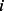 *on the function* 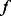 *is defined as*

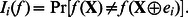



The influence can be related to the Fourier spectra as follows [26]:
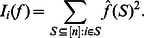



The average sensitivity is a measure to quantify the impact of a random perturbation of the inputs of a Boolean function. It is defined as the sum of the influences of all input variables of 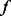.

**Definition 6** *(* *[19]*, *[24]* *) The average sensitivity of* 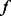 *is defined as*

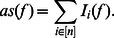



Consequently the average sensitivity can also be expressed in terms of the Fourier coefficients [24] as:
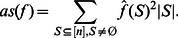
(11)


#### Restricted functions

To investigate the average sensitivity of restricted functions we first define 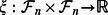 by.
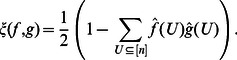
(12)


Our next result shows the relation between the average sensitivity of a BF and the average sensitivity of its two restricted functions.

**Theorem 1** *Let* 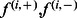 *be the restrictions of* 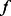 to some relevant variable 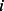 *of* 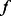 *. Then*

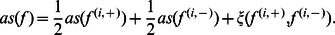



*Proof.* Starting from Eq. (11), we can fractionize the Fourier coefficients according to Proposition 3. This yields:
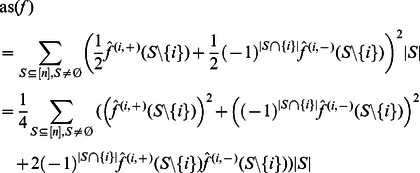
which leads us to:



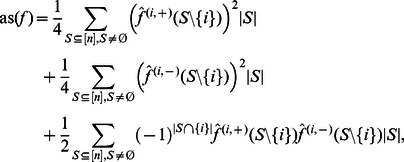
and hence to:



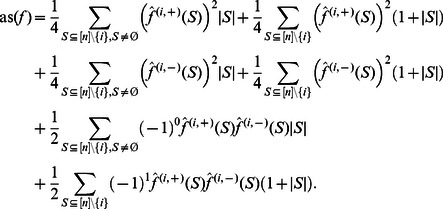





Since 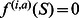 for all 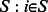, we can write
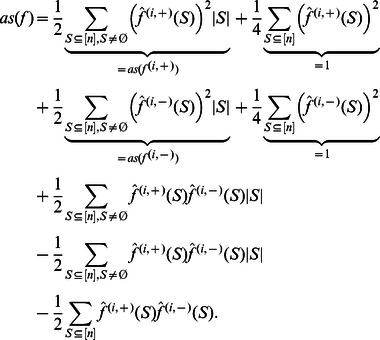



Finally we get.
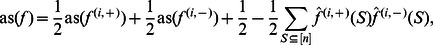
which concludes the proof.

For NCFs we obtain:

**Corollary 6** *The average sensitivity of a* 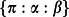 *NCF can recursively be described as:*

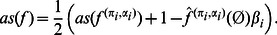
(13)


In [21] an upper bound on the average sensitivity of NCF has been conjectured. In the following theorem, we prove this conjecture to be correct.

**Theorem 2** *The average sensitivity of a NCF with* 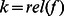 *relevant and uniformly distributed variables is bounded by*

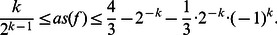
(14)


The bounds in Eq. (14) will turn out to be tight.

*Proof*. We first prove the upper bound in Eq. (14). Let us recall Corollary 6:
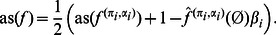
(15)


If we apply Corollary 6 again on 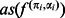 and use Corollary 5 on 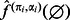, Eq. (15) becomes:
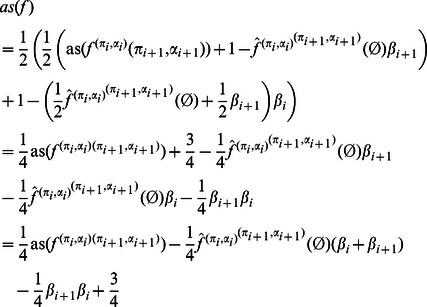





Since 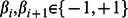 and 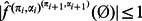,
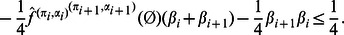



Thus we obtain
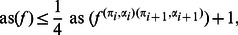
(16)where 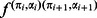 has 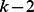 relevant variables. We will now show the theorem by induction. For 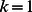 the upper bound in Eq. (14) simplifies to



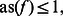
which is obviously true by definition. For 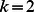 the upper bound in Eq. (14) results in



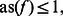
which is also true and can be verified by inspecting all possible functions.

Using Eq. (14) as the induction hypothesis, and applying it on 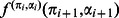 in Eq. (16), which has 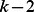 relevant variables, yields:
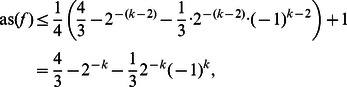
which concludes the induction.

The lower bound in Eq. (14) can be proven along the lines of the proof of the upper bound, using the following inequality, which follows from Corollary 6 and Proposition 6:
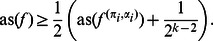



The tightness of the bounds in Eq. (14) is shown in Propositions 8 and 9.

We can further upper bound the right hand side of Theorem 2 in order to make it independent of the number of relevant variables 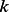:

**Corollary 7** *The average sensitivity of a NCF with uniformly distributed variables satisfies*

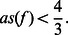



We next show that the bounds in Theorem 2 are tight.

**Proposition 8** *Let* 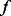 *be a NCF, whose variables are all most dominant. Then* 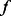 *satisfies the upper bound in Theorem 2 with equality.*

*Proof.* Starting from Corollary 6 and using that, by Corollary 3, 
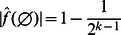
 and 
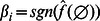
 for all 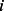, we get:
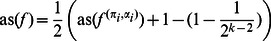
(17)

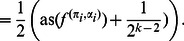
(18)


Since 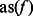 depends on 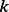 relevant variables, while 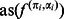 depends only on 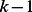 relevant variables, Eq. (17) becomes:
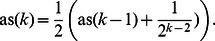



The proof is concluded by solving this recursion using induction.

**Proposition 9** *Let* 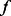 *be a NCF with alternating* 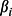 *, i.e.,* 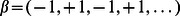 *or* 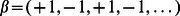. *Then* 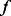 *fulfills the upper bound in Eq. (14) of Theorem 2 with equality.*

*Proof.* Similar to the proof of the previous proposition we start from Corollary 6 and use 
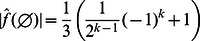
. The proof is established by solving the recursion.

Propositions 8 and 9 show that the maximal and minimal average sensitivity is achieved, if the absolute value of the zero coefficient is minimal and maximal, respectively. The following proposition gives a bound on the average sensitivity for fixed 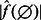.

**Proposition 10** *Let* 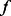 *be a NCF with uniform distributed inputs. Then*

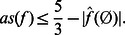



*Proof.* Combining Corollaries 6 and 7, we get:
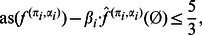
and since 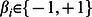:



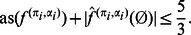



Substituting 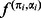 by 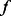 concludes the proof.

## Discussion

**Figure 1:**
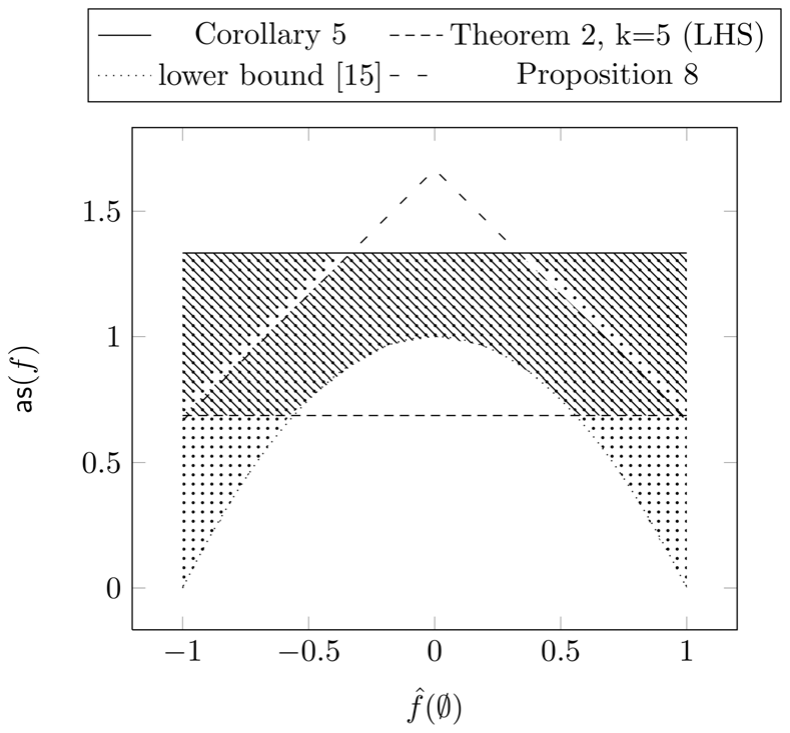
Bounds on the average sensitivity. The dotted-area corresponds to the possible values for the average sensitivity of a NCF, the lined area to BFs with 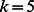 input variables.

In Figure 1 we summarize the bounds from the previous section. Specifically, we plot the average sensitivity versus the zero coefficient. Additionally, we include a lower bound on the average sensitivity that is independent of the number of relevant variables and applies for any 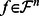 and can be found in [27]. One can see that this bound intersects with our lower bound (which we plotted for 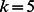), though we stated that our bound is tight. However, this is not a contradiction, since the lower bound in Theorem 2 is achieved for functions with large absolute zero coefficients, which are located outside the intersection.

For 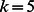 our lower bound forms a triangle with the upper bound as formulated in Proposition 10. The NCFs with all variables being most dominant are located in the left and right corners of that triangle. However the lower bound decreases in 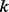 and with it the most dominant NCFs.

The upper bound in Corollary 7 also intersects with the bound of Proposition 10. Again, this is not a contradiction, since NCFs reach this bound only for small absolute zero coefficients.

In general the average sensitivity is upper bounded by 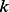, i.e., 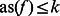. As shown in [28] for monotone and in [15] for unate, i.e., locally monotone, functions, the average sensitivity is upper bounded by 
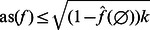
. This bound is tight up to a multiplicative constant, see e.g. [29]. A function is unate, if it is monotone in each variable. In a regulatory network, where each regulator acts either inhibitory or exhibitory towards a certain gene, each function is unate. NCFs form a subclass of unate functions. Thus, our results show, that even within the class of unate functions, the average sensitivity of NCFs is remarkably low. Since a low average sensitivity has a positive effect on the stability of Boolean networks [2], our result gives an explanation for the remarkable stability of BNs with NCFs.

## Conclusion

In this paper we investigated canalizing and nested canalizing Boolean functions using Fourier analysis. We gave recursive representations for the zero coefficient and the average sensitivity based on the concept of restricted BFs.

We addressed the average sensitivity of nested canalizing functions and provided a tight upper and lower bound on the average sensitivity. We showed that the lower bound is achieved by functions whose input variables are all most dominant and which maximize the absolute zero coefficient. The upper bound is reached by functions, whose canalized values are alternating.

We provided an upper bound on the average sensitivity, namely 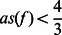, which has been conjectured in literature [21]. Finally, we derived a bound on the absolute zero coefficient and the average sensitivity and discussed the stabilizing effect of nested canalizing functions on the network dynamics.

It is worth noting that all those results rely on the assumption of uniformly distributed inputs. This rises the question, if the results can be generalized to other distributions. The recursive representations can easily be extended to product distributed input variables. But without further constraints there always exists a distribution, which maximizes the average sensitivity, i.e., for any function with 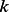 relevant variables the average sensitivity can be 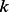.
